# Multimorbidity: can general practitioners identify the health conditions most important to their patients? Results from a national cross-sectional study in Switzerland

**DOI:** 10.1186/s12875-018-0757-y

**Published:** 2018-05-17

**Authors:** Anouk Déruaz-Luyet, Alexandra A. N’Goran, Jérôme Pasquier, Bernard Burnand, Patrick Bodenmann, Stefan Zechmann, Stefan Neuner-Jehle, Nicolas Senn, Daniel Widmer, Sven Streit, Andreas Zeller, Dagmar M. Haller, Lilli Herzig

**Affiliations:** 1 Institute of Family Medicine, University of Lausanne, 44 rue du Bugnon, 1011 Lausanne, Switzerland; 2 Institute of Social and Preventive Medicine, Lausanne University Hospital, Lausanne, Switzerland; 3 Department of Ambulatory Care and Community Medicine, Lausanne University Hospital, Lausanne, Switzerland; 4 Institute of Primary Care, University of Zurich, Zurich, Switzerland; 5 Institute of Primary Health Care (BIHAM), University of Berne, Berne, Switzerland; 6 Centre for Primary Health Care, University of Basel, Basel, Switzerland; 7 Primary Care Unit, Faculty of Medicine, University of Geneva, Geneva, Switzerland

**Keywords:** Multimorbidity, Family medicine, Patient–provider agreement

## Abstract

**Background:**

Faced with patients suffering from more than one chronic condition, or multimorbidity, general practitioners (GPs) must establish diagnostic and treatment priorities. Patients also set their own priorities to handle the everyday burdens associated with their multimorbidity and these may be different from the priorities established by their GP. A shared patient–GP agenda, driven by knowledge of each other’s priorities, would seem central to managing patients with multimorbidity. We evaluated GPs’ ability to identify the health condition most important to their patients.

**Methods:**

Data on 888 patients were collected as part of a cross-sectional Swiss study on multimorbidity in family medicine. For the main analyses on patients-GP agreement, data from 572 of these patients could be included. GPs were asked to identify the two conditions which their patient considered most important, and we tested whether either of them agreed with the condition mentioned as most important by the patient. In the main analysis, we studied the agreement rate between GPs and patients by grouping items medically-related into 46 groups of conditions. Socio-demographic and clinical variables were fitted into univariate and multivariate models.

**Results:**

In 54.9% of cases, GPs were able to identify the health condition most important to the patient. In the multivariate model, the only variable significantly associated with patient–GP agreement was the number of chronic conditions: the higher the number of conditions, the less likely the agreement.

**Conclusion:**

GPs were able to correctly identify the health condition most important to their patients in half of the cases. It therefore seems important that GPs learn how to better adapt treatment targets and priorities by taking patients’ perspectives into account.

**Electronic supplementary material:**

The online version of this article (10.1186/s12875-018-0757-y) contains supplementary material, which is available to authorized users.

## Background

Multimorbidity is defined as the co-occurrence of two (or three) or more chronic health conditions in one person without any condition being considered an index condition [1]. Faced with patients with multimorbidity, GPs must often set priorities between the different chronic conditions, as managing them all is often infeasible in everyday practice. Patients also set their own disease and treatment priorities to handle the everyday burdens associated with their multimorbidity and these may be different from the priorities established by their GP.

Evidence shows that good communication between patients and GPs leads to more agreement between them and a better care outcome [2]. However, studies examining the match between physicians’ and patients’ priority health conditions have produced contrasting results, and most of these studies were conducted in diabetic patients [3–5]. High levels of agreement on which conditions were considered important from patients’ and GPs’ perspectives have been reported previously, but levels of agreement were lower in patients reporting a poor health status [5]. A common patient–GP (or patient–provider) agenda is key to a successful therapeutic process, particularly in patients with multimorbidity, and this is generally dependent on good patient–GP communication and shared decision-making [2, 6, 7]. Our hypothesis was that more experienced GPs and GPs with a sub-specialization in psychosocial and psychosomatic medicine, based on the development of strong communication, would be positively associated with agreement between GPs and patients, as would a higher number of visits to the GP in the last 12 months and more years of follow-up by the same GP. In contrast, a high number of chronic conditions and a greater number of medical doctors involved in the patient’s treatment could be a reflection of more complicated cases or sicker patients, for whom setting priorities would be more difficult. Lower health-literacy and less well-educated patients could also be factors complicating patient–GP communication and could be negatively associated with agreement rates between GPs and patients. The presumed direction of the association between the number of consultations handled by the GP each week was less clear-cut, however, as more consultations per week could mean either greater knowledge of each patient with multimorbidity or less time spent per consultation.

Studies to date in primary care settings have reported low levels of agreement between patients and GPs on health and treatment priorities [8]. Qualitative studies have underlined that they do not seem to give the same level importance to the same conditions, and to some extent, GPs appear unaware of their patients’ priorities [8–10]. Voigt et al. [8] showed that even if GPs were able to identify the number of health problems that their patients considered important, they did not give the same level of importance to the same conditions. Patients’ ratings of health conditions have been shown to be related to their current experience of their condition, whereas GPs’ ratings were more related to the condition’s prognosis [9]. Thus, our aim was to evaluate whether GPs could identify the condition that their patients with multimorbidity considered most important. To the best of our knowledge, this is one of the first quantitative study addressing this topic.

## Methods

We analyzed data from the Multimorbidity in Family Medicine study, a cross-sectional study in Switzerland. The study protocol and a detailed description of the database established have been published elsewhere [11, 12].

### Setting and participants

Data collection took place in Switzerland between January and September 2015. A convenience sample of 100 GPs randomly enrolled patients during scheduled consultations at their private practices. Each GP was provided with a randomisation calendar specifying which patients to enrol on each half-day during the recruitment weeks. All multimorbid patients above 18 years old, followed by their GP for at least 6 months and suffering from at least three of the 75 chronic conditions on a predefined list based on the International Classification of Primary Care 2 (ICPC 2) were considered eligible [13, 14] and signed a written consent to participate to the study. GPs completed a paper-based questionnaire for each included patient and patients enrolled completed a telephone-based questionnaire. Only anonymous dates were transmitted to the research team. The study protocol (n° 314/15) was approved by the Human Research Ethics Committee of the Canton Vaud.

### Variables

#### Outcome variable

We evaluated the agreement between what patients considered to be their most important health condition and what GPs thought patients considered to be their most important health condition. Conditions were considered to be “in agreement” if one of two conditions considered important by the GP belonged to the same group of conditions as the one considered most important by the patient; they were “in disagreement” otherwise. Agreement between GPs and patients was considered as a dichotomous variable.

#### Independent variables

The GP-related variables of interest were: sex, number of years of practice, number of consultations per week, practice location, and a sub-specialization in psychosocial and psychosomatic medicine, i.e., a postgraduate qualification to handle psychosomatic and psychosocial factors influencing the development and management of diseases. The patient-related variables were: age, sex, number of chronic conditions, level of education, number of medical doctors involved in the patient’s treatment, number of visits to the GP in the last 12 months, and number of years with this GP.

### Data sources

#### GP level

We asked the GPs to list the two health conditions they thought were most important to their patients.

#### Patient level

During a telephone interview, patients were asked, “If you had the power to heal one of your medical problems, which one would you choose first?” In the absence of a validated question on this topic, we designed this question during several internal consensus discussions. Although it did not precisely match the GPs’ question, it could be answered by patients with lower levels of health literacy. In line with the study’s protocol and its overall goals, interviewers were trained to conduct standardized telephone interviews. As a result, they did not to attempt to establish more precise patient answers when they might have been needed.

### Measurement

Only answers that could be transferred into a corresponding ICPC-2 code were used. In the knowledge that patients and GPs may refer to the same conditions differently and with different levels of precision, we grouped related chronic conditions for agreement analyses. For the main analysis, we considered a classification involving 46 groups of diseases derived from the classification developed by van den Bussche et al. for elderly patients with multimorbidity and subsequently adapted specifically to our assessment by three members of the research team [15]. (Additional file 1: Table S1). We evaluated agreement between GPs’ and patients’ answers based on these 46 groups. To avoid over-interpretation, for both analyses, any responses that could not be unequivocally attributed to one group were considered as missing data. In a sensitivity analysis, we used fifteen chapters of the ICPC-2 classification to group items (General and unspecified; Blood/blood-forming organs and immune system; Digestive; Eye; Ear; Cardiovascular; Musculoskeletal; Neurological; Psychological; Respiratory; Skin; Endocrine/metabolic and nutritional; Urological; Female Genital; Male Genital). We excluded the two chapters on pregnancy and social aspects, which did not encompass chronic conditions per se [16]. We also considered answers in which more than one code was given, as follows. If all of the codes belonged to the same group or chapter, we attributed the condition to that group or chapter, respectively. If the codes did not belong to the same group or chapter, or if the group or chapter could not be clearly defined, the codes were considered as missing data.

Agreement was considered a binary outcome. Agreement occurred when the most important condition identified by the patient matched one of the two conditions given by the GP. The independent variables of number of years of practice, number of years of follow-up by the GP, the patient’s age, and the number of visits to the GP in the last 12 months were considered as continuous variables. All other variables were considered as categorical variables. The different numbers of chronic conditions were stratified into three groups: “3”, “4 or 5”, and “6 or more” chronic conditions. This categorization was made according to the levels of multimorbidity defined by Kadam et al. [17]

### Study size

In all, 888 patients were included in the cross-sectional study. After discarding patients with missing values for the matching variables, 841 patients remained. From these patients, we excluded those with ambiguous responses, i.e. responses that could not easily be assigned to one category. As a result, 579 and 593 were retained for the main and sensitivity analyses, respectively. We eliminated patients with incomplete data for any of the independent variables considered. Finally, 572 and 585 patients (64.4 and 65.9% of the original sample) were included in the main and sensitivity analyses, respectively. Patients discarded due to missing data were not different from the patients included in the main analysis in terms of sex (chi^2^, 1 df, *p* = 0.05), number of chronic conditions (chi^2^, 2 df, *p* = 0.45), level of education (chi^2^, 6 df, *p* = 0.25), number of MDs involved in patient’s treatment (chi^2^, 3 df, *p* = 0.24), number of GP visits in last 12 months (t-test, *p* = 0.86). They were slightly older than patients included (mean age (SD): 74.3 (11.5) versus 72.2 (12.2), t-test *p* < 0.05).

### Analyses

For both analyses, we examined whether one of the two conditions identified by the GP matched the most important condition identified by the patient. For the main analysis, agreement was evaluated with regards to 46 groups of conditions (Additional file 1: Table S1), whereas for the sensitivity analysis this was done with regards to 15 chapters of the ICPC-2 classification.

### Statistical methods

We conducted bivariate and multivariate logistic regressions to evaluate the associations between GP-related and patient-related variables with the agreement variable and to evaluate any potential clustering effects around GPs’ variables.

## Results

Agreements between the most important health condition for patients and those perceived to be the most important to patients by GPs are reported in Table 1. In the main analysis, agreement occurred in 54.9% of cases (*N* = 314). For the sensitivity analysis, agreement occurred in 63.8% of the cases (*N* = 373) (Table 1). No clustering effects around GPs’ variables were detected.

**Table 1:** Patient–GP rates of agreement on the condition ranked as most important to the patient (data collection January–September 2015)

		46 groups of conditions (*N* = 572) N (%)		15 chapters of the ICPC-2 (*N* = 585) N (%)	
Agreement	The condition that the GP thinks is most important to the patient belongs to the same group/chapter as the condition considered most important by the patient.	217 (37.9)	314 (54.9)	260 (44.4)	373 (63.8)
	The condition that the GP thinks is the second most important to the patient belongs to the same group/chapter as the condition considered most important by the patient.	97 (17)		113 (19.3)	
Disagreement	The conditions that the GP thinks are most important to the patient do not belong to the same group/chapter as the condition considered most important by the patient.	258 (45.1)		212 (36.2)	

In the main model based on the classification of items in 46 groups, only the number of chronic conditions was significantly associated with the outcome both in univariate (Table 2) and multivariate analyses (Table 3).

**Table 2:** Bivariate analyses of all the variables considered in the study (Data collection January–September 2015)

		46 groups of conditions (*N* = 572)			15 chapters of the ICPC-2 (*N* = 585)		
		No	Yes	*p*-value	No	Yes	*p*-value
Patient-related variables							
No. of chronic conditions^+^	3, N (%)	31 (12)	59 (18.8)	0.06	26 (12.3)	66 (17.7)	0.20
	4 or 5	121 (46.9)	125 (39.8)		98 (46.2)	154 (41.3)	
	6 or more	106 (41.1)	130 (41.4)		88 (41.5)	153 (41)	
Age^++^	Mean, years (SD)	73.1 (12.1)	71.5 (12.3)	0.11	73 (11.5)	71.9 (12.7)	0.28
Sex^+^	Male, N (%)	117 (45.3)	144 (45.9)	0.93	101 (47.6)	171 (45.8)	0.73
	Female	141 (54.7)	170 (54.1)		111 (52.4)	202 (54.2)	
Level of education+	Primary school/no diploma, N (%)	43 (16.7)	42 (13.4)	0.28	36 (17)	51 (13.7)	0.82
	Secondary school	20 (7.8)	24 (7.6)		16 (7.5)	28 (7.5)	
	Practical professional training	61 (23.6)	86 (27.4)		56 (26.4)	94 (25.2)	
	High school or equivalent	33 (12.8)	33 (10.5)		23 (10.8)	45 (12.1)	
	Vocational school or training	58 (22.5)	92 (29.3)		51 (24.1)	105 (28.2)	
	Non-university higher education	26 (10.1)	24 (7.6)		17 (8)	33 (8.8)	
	University	17 (6.6)	13 (4.1)		13 (6.1)	17 (4.6)	
No. of MDs involved in patient’s treatment^+^	1, N (%)	73 (28.3)	81 (25.8)	0.81	58 (27.4)	98 (26.3)	0.75
	2	76 (29.5)	89 (28.3)		57 (26.9)	112 (30)	
	3	54 (20.9)	75 (23.9)		48 (22.6)	88 (23.6)	
	4 or more	55 (21.3)	69 (22)		49 (23.1)	75 (20.1)	
No. of GP visits in last 12 months^++^	Mean, N (SD)	12.9 (8.9)	13 (8.7)	0.87	13.2 (9.3)	13.2 (9.2)	0.95
No. of years followed by GP^++^	Mean, years (SD)	10.9 (7.9)	11.2 (8.5)	0.59	10.4 (7.9)	11.5 (8.4)	0.13
GP-related variables							
Sex+	Male, N (%)	196 (76)	220 (70.1)	0.13	152 (71.7)	271 (72.7)	0.85
	Female	62 (24)	94 (29.9)		60 (28.3)	102 (27.3)	
No. of years of practice++	Mean, years (SD)	17.3 (9.6)	18 (9.6)	0.42	16.5 (9.5)	18.2 (9.5)	0.03
No. of consultations per week+	≤ 30 N (%)	2 (0.8)	3 (1)	0.82	1 (0.5)	4 (1.1)	0.59
	]30–70]	72 (27.9)	84 (26.8)		60 (28.3)	96 (25.7)	
	]70–110]	117 (45.3)	148 (47.1)		100 (47.2)	176 (47.2)	
	]110–170]	59 (22.9)	64 (20.4)		46 (21.7)	79 (21.2)	
	> 170	8 (3.1)	15 (4.8)		5 (2.4)	18 (4.8)	
Practice location+	Urban, N (%)	109 (42.2)	136 (43.3)	0.97	92 (43.4)	157 (42.1)	0.86
	Suburban	108 (41.9)	129 (41.1)		85 (40.1)	158 (42.4)	
	Rural	41 (15.9)	49 (15.6)		35 (16.5)	58 (15.5)	
Sub-specialization in psychosocial & psychosomatic medicine	No, N (%)	236 (91.5)	294 (93.6)	0.34	198 (93.4)	347 (93)	1.00
	Yes	22 (8.5)	20 (6.4)		14 (6.6)	26 (7)	

Agreement is positive if one of the two conditions the GP considered as important to the patient belongs to the same category as the condition considered most important by the patient. Otherwise, it is negative. Difference between groups tested using: + Fisher test; ++ t-test; practice location was evaluated using zip codes and data obtained from the Swiss Federal Statistical Office

**Table 3:** - Multivariate analyses of all the variables considered in the study (Data collection January–September 2015)

		46 groups of conditions (N = 572)		15 chapters of ICPC-2 (N = 585)	
		Odds ratio (95% CI)	*p*-value	Odds ratio (95% CI)	*p*-value
Patient-related variables					
No. of chronic conditions	3 (reference)				
	4 or 5	0.53 (0.31;0.89)	0.02	0.6 (0.34;1.01)	0.06
	6 or more	0.59 (0.34;1)	0.05	0.64 (0.36;1.09)	0.11
Age	per year	0.99 (0.98;1.01)	0.22	0.99 (0.98;1.01)	0.32
Sex	Male (reference)				
	Female	0.98 (0.69;1.41)	0.92	1.11 (0.77;1.59)	0.59
Level of education	Primary school/ no diploma (reference)				
	Secondary school	1.17 (0.55;2.51)	0.68	1.18 (0.55;2.58)	0.68
	Practical professional training	1.53 (0.87;2.69)	0.14	1.16 (0.66;2.03)	0.60
	High school or equivalent	1.05 (0.54;2.05)	0.89	1.33 (0.68;2.65)	0.41
	Vocational school or training	1.66 (0.94;2.94)	0.08	1.45 (0.82;2.57)	0.20
	Non-university higher education	0.89 (0.42;1.89)	0.76	1.28 (0.59;2.8)	0.54
	University	0.79 (0.32;1.92)	0.60	0.91 (0.37;2.25)	0.84
No. of MDs involved in patient’s treatment	1 (reference)				
	2	1.05 (0.66;1.67)	0.84	1.13 (0.7;1.82)	0.62
	3	1.22 (0.74;2.01)	0.44	1.07 (0.64;1.78)	0.80
	4 or more	1.11 (0.67;1.84)	0.69	0.87 (0.52;1.45)	0.59
No. of GP visits in last 12 months	per unit	0 (0;0)		0 (0;0)	
No. of years followed by GP	per year	1 (0.97;1.03)	0.92	1 (0.97;1.03)	0.95
GP-related variables					
Sex	Male (reference)				
	Female	1.49 (0.96;2.33)	0.08	0.99 (0.64;1.54)	0.96
No. of years of practice	per year	1.01 (0.99;1.04)	0.39	1.02 (0.99;1.05)	0.20
No. of consultations per week	≤ 30 (reference)				
	]30–70]	0.82 (0.1;5.49)	0.83	0.43 (0.02;3.26)	0.47
	]70–110]	1.01 (0.12;7.01)	0.99	0.43 (0.02;3.36)	0.48
	]110–170]	0.92 (0.11;6.56)	0.94	0.42 (0.02;3.38)	0.47
	> 170	1.36 (0.14;11.61)	0.78	0.78 (0.03;8.1)	0.85
Practice location	Urban (reference)				
	Suburban	0.92 (0.62;1.36)	0.68	1.08 (0.73;1.61)	0.70
	Rural	0.92 (0.54;1.59)	0.77	1 (0.58;1.73)	0.99
Sub-specialization in psychosocial & psychosomatic medicine	No (reference)				
	Yes	0.76 (0.38;1.49)	0.42	1.08 (0.53;2.26)	0.84

More precisely, agreement between GPs and patients decreased as the number of chronic conditions increased. Compared to “3” chronic conditions, the odds ratios (95% confidence interval, CI) for agreement between when there were “4 or 5” and “6 or more” chronic conditions were 0.53 (95% CI, 0.31–0.89) and 0.59 (95% CI, 0.34–1), respectively. Thus, there was only a statistically significant difference in agreement when the “4 or 5” conditions group was compared to the “3” chronic conditions group. No cluster effects were observed around any of the GPs, and the rates of agreement were similar for all the GPs enrolled. Rates of agreement were 65.6, 50.8, and 55.1% for the “3”, “4 or 5”, and “6 or more” chronic conditions groups, respectively (Table 4).

**Table 4:** Rates of agreement in relation to the number of chronic conditions, split into 46 groups and 15 chapters of the ICPC-2

Number of chronic conditions	Agreement on one of the 46 groups of conditions – main analysis (N = 572)				Agreement on one of the 15 chapters of the ICPC-2 classification – sensitivity analysis (N = 585)			
	No agreement		Agreement		No agreement		Agreement	
3	31	34.4%	59	65.6%	26	28.3%	66	71.7%
4 or 5	121	49.2%	125	50.8%	98	38.9%	154	61.1%
6 or more	106	44.9%	130	55.1%	88	36.5%	153	63.5%

The groups of conditions most frequently mentioned by patients as the most important conditions included Musculoskeletal conditions and pain and Spine conditions/back pain (groups 18, 19). For these two groups of conditions, rates of agreement between GPs and patients were 67.8 and 42.9%, respectively (Additional file 2: Table S2 a). Unfortunately for this analysis, the limited number of observations in some groups of conditions limited the interpretation of results.

The sensitivity analysis conducted on broader groups of conditions relating to chapters if the ICPC-2 showed similar results. Compared to “3” chronic conditions, the odds ratio for agreement between when there were “4 or 5” and “6 or more” chronic conditions were 0.6 (95% CI, 0.34–1.01) and 0.64 (95% CI, 0.36–1.09), respectively (Additional file 2: Table S2 b).

## Discussion

When chronic conditions were classified into 46 categories, GPs were able to identify the condition most important to their patients in 54.9% of cases. The rate of agreement was higher (63.8%) when looking for agreement with items grouped in the chapters of the ICPC-2 classification. Differences between the perspectives of GPs and patients have previously been shown for such aspects of disease management as the cure or relief of symptoms [18], disease severity [19], and the level of suffering [20]. In the present study, back pain and musculoskeletal conditions, often symptomatic, were the conditions most often ranked first by patients. However, agreement with GPs on these conditions was contrasted, being higher than average for musculoskeletal conditions but lower for spine and back syndromes. Any interpretation of these analyses is limited by the reduced number of conditions per group. Nevertheless, these results seem to confirm that pain and symptomatic chronic conditions are patients’ first priorities, whereas GPs’ priorities may be more prognosis-based [7].

The only variable associated with a difference in the rates of agreement between GPs and patients was the number of chronic conditions. This was expected because the probability of patient–GP agreement is higher in patients with fewer health conditions [5, 6]. In the present study, however, the rate of agreement for patients suffering from 4 or 5 chronic conditions was lower than for patients with 3 chronic conditions, but the rate did not decrease further for patients suffering from 6 or more. This suggests that patient–GP agreement may depend somewhat on factors other than those evaluated in this study. Similarly to our results, a previous study showed that a GP’s knowledge of a patient’s health was not influenced by the patient’s age, level of education, number of consultations in the previous 6 months or the duration of the patient–GP relationship [6]. Although the present study did not report the GP’s sex, this did not seem to be associated with agreement on important health conditions [5].

Our study failed to show any positive associations between the rate of patient–GP agreement and a sub-specialization in psychosocial or psychosomatic medicine, a longer follow-up, or more frequent appointments. This was despite our anticipation that these factors would have given GPs greater knowledge of their patients. An alternative explanation could be that patients with more frequent appointments and longer follow-up suffer from more chronic conditions and are more complicated for GPs to handle.

Our study has some limitations. To identify the health conditions most important to patients, we asked them which of their chronic health problems they would like to heal first and second, given the opportunity. Although admittedly the question was not formally validated, both GPs and patients deemed the question appropriate during a pilot phase But, it slightly differed from the question asked to GPs. Using the term “healing” with patients might have encouraged them to list their most symptomatic conditions. But, as previous studies showed that in any case patients with multimorbidity generally prioritized the treatment of symptomatic diseases over asymptomatic diseases [5, 21, 22] we do not believe that this could have biased our study’s outcome to a great extent. We also did not evaluate the importance of certain other factors previously identified as influencing patients’ prioritization of health problems, such as emotional disorders interfering with day-to-day activities and perceived unfavorable diagnoses [9]. And we did not include detailed information about duration, severity, treatment side effects, or functional impairments linked to each condition. Finally, patients’ priorities regarding multiple chronic conditions are likely to change over time in response to factors such as a worsening of one of the conditions [23], the negative effects of a treatment [24], or contact with health professionals [23, 24] and should be re-examined whenever the patient’s clinical status changes [4].

An alternative explanation for the absence of positive associations between any of the patient or GP variables considered and the rate of patient-GP agreement could be related to a lack of patient-centered care. Indeed, patient-centered care could be a more holistic or better overall way of treating patients with multimorbidity; one more centered on general priorities such as staying alive, maintaining independence, reducing/eliminating pain, and reducing/eliminating symptoms rather than on treating specific health conditions or diseases. A study evaluating patients’ prioritization of these outcomes reported that 76% ranked maintaining independence as the most important factor; this was followed by the relief of symptoms [25]. In another study of patients with multimorbidity, of those who ranked staying alive as their primary concern (11%), 66% ranked maintaining independence as their secondary concern [25].

## Conclusion

The present national, cross-sectional study of multimorbidity in Switzerland is one of the first evaluations of GPs’ ability to identify the chronic health condition that their patients consider most important. We showed that in more than half of the cases examined, GPs were able to identify this condition, as referenced in a classification of 46 groups of conditions. This rose to about two thirds of cases when the conditions were grouped by chapter in the ICPC-2 classification. In view of these results, it seems crucial that GPs evaluate, which conditions are most important to their patients and subsequently adapt treatment targets and priorities accordingly. Good communication and patient–provider agreement would seem to be essential in this regard. Further research is needed to understand the factors underlying rates of agreement between GPs and patients, as well as which aspects—specific health conditions and symptoms or a more comprehensive or alternately holistic treatment method—should be at the heart of the patient-centered care for patients with multimorbidity.

## Additional files

Additional file 1: **Table S1.** Classification of items of the ICPC-2 into 46 categories adapted from van den Bussche et al. (DOCX 25 kb)

Additional file 2: **Table S2.** a Agreement as per the 46 groups of conditions (*N* = 572). **Table S2.** b Agreement as per the chapters of the ICPC-2 classification (*N* = 585). (DOCX 20 kb)

## Abbreviation

GP: General practitioners
