# Supplementary material for: Multimorbidity: can general practitioners identify the health conditions most important to their patients? Results from a national cross-sectional study in Switzerland
Source: BMC Fam Pract. 2018 May 17;19:66. doi: 10.1186/s12875-018-0757-y (PMC5960174; doi:10.1186/s12875-018-0757-y)
Supplement: Supplementary file 1 — Table S1. Classification of items of the ICPC-2 into 46 categories adapted from van den Bussche et al. (DOCX 25 kb) [file 12875_2018_757_MOESM1_ESM.docx]

Supplementary Table 1 - Classification of items of the ICPC-2 into 46 categories adapted from van den Bussche et al.,

|  | **Description** | **ICPC-2 codes** | **van den Bussche (ICD)** |
| --- | --- | --- | --- |
| 1 | Weakness, neurasthenia/overwork, other | A04, A06, A28, A29, A82, A85, A88, A99, K88, P78 | Does not exist |
| 2 | Tuberculosis, malaria, mononucleosis, viral disease, AIDS, sexually transmitted diseases | A70, A73, A75, A77, A78, B90, D70, D72, X70, X71, X72, X73, X74, X90, X91, X92, Y70, Y71, Y72, Y73, Y74, Y75, Y76 | Does not exist |
| 3 | Cancer | A21, A79, B72, B73, B74, D74, D75, D76, D77, D78, F74, H75, K72, L71, N74, N75, N76, R84, R85, R86, S77, S78, S79, S80, T71, T72, T73, U75, U76, U77, X75, X76, X77, X78, X79, Y77, Y78, Y79 | Group 40 (cancer) C00-C14, |
| 4 | Anemia, iron deficiency, vit. B12 deficiency, other splenomegaly | B04, B28, B29, B78, B79, B80, B81, B82, B83, B87, B99, A10 | Group 35 Anemia |
| 5 | Abdominal pain, cramps, epigastric pain, nausea, vomiting, hematemesis, melena, teeth/mouth symptoms, swallowing, esophagus, duodenal ulcer, hernia: inguinal, hiatus | D01, D02 D03, D04, D07, D09 D10, D14, D15, D19, D20, D21, D29, D80, D82, D83, D84, D85, D86, D87, D89, D90, D91 | Group 42 Chronic gastritis/GERD |
| 6 | Digestive general, bloating, diarrhea, constipation, incontinences, diverticular, irritable bowel, chronic enteritis, hemorrhoids | D08, D11, D12, D16, D17, D18, D92, D93, D94, D95, D99, T91, K96 | Group 26 Hemorrhoids  Group 27 Intestinal diverticulosis |
| 7 | Liver | D13, D23, D97, D98 | Group 14 Liver disease  Group 24 Chronic cholecystitis/Gallstones |
| 8 | Eye | F01, F05, F13, F14, F16, F17, F18, F28, F29, F83, F84, F85, F86, F91, F92, F93, F94, F95, F99 | Group 4 Severe vision reduction |
| 9 | Ear | H01, H02, H03, H74, H83, H84, H86, H99 | Group 23 Severe hearing loss |
| 10 | Cardiovascular peripheral, phlebitis, varicose veins | K06, K07, A08, K94, K95 | Group 13 Lower limb varicosis |
| 11 | Risk factor cardiovascular disease, lipid disorder | K22, T93 | Group 2 Lipid metabolism disorders |
| 12 | Ischemic heart diseases | K01, K02, K03, K74, K75, K76, | Group 7 Chronic ischemic heart disease |
| 13 | Heart failure, pulmonary heart, other | K28, K73, K77, K82, K84, K99 | Group 22 Cardiac insufficiency |
| 14 | Arrhythmia | K04, K05, K78, K79, K80 | Group 9 Cardiac arrhythmias |
| 15 | Valve disease | K83 | Group 29 Cardiac valve disorders |
| 16 | Hypertension | K85, K86, K87 | Group 1Hypertension |
| 17 | Cerebrovascular disease | K89, K90, K91, K92, N18, N19, N28, N29 | Group 18 Atherosclerosis/PAOD  Group 21 Cerebral ischemia/Chronic  stroke |
| 18 | Musculoskeletal (arm and legs), pain | L05, L07, L08, L09, L10, L11, L12, L13, L14, L15, L16, L17, L18, L19, L20, L28, L70, L75, L76, L89, L90, L91, L92, L93, L98, L99, A01 | Group 3 Chronic low back pain |
| 19 | Spine | L01, L02, L03, L04, L83, L84, L85, L86 | Group 3 Chronic low back pain |
| 20 | Osteoporosis | L94, L95 | Group 19 Osteoporosis |
| 21 | Arthritis, rheumatoid, gout | L87, L88, T92 | Group 28 Rheumatoid arthritis/Chronic  polyarthritis |
| 22 | Headache | N01, N03, N89, N90, N91, N92, N95 | Group 38 Migraine/Chronic headache |
| 23 | Epilepsy | N07, N88 | Does not exist |
| 24 | Parkinson’s | N87 | Group 39 Parkinson’s disease |
| 25 | Vertigo | N17, H82 | Group 31 Dizziness |
| 26 | Peripheral neuritis, restless legs, other neuropathies | N04, N05, N06, N08, N16, N93, N94, N99 | Group 30 Neuropathies |
| 27 | Multiple sclerosis, poliomyelitis | N70, N86 | Does not exist |
| 28 | Anxiety, depression, other psychological troubles, social problems | A25, A26, P01, P02, P03, P04, P10, P29, P74, P75, P76, P77, P79, P82, Z14, Z15, Z28, Z29 | Group 15 Depression  Group 25 Somatoform disorders  Group 36 Anxiety |
| 29 | Sleep disturbance | P06 | Group 44 Insomnia |
| 30 | Abuse of alcohol, medication, drugs | P15, P18, P19 | Does not exist |
| 31 | Tobacco abuse | P17 | Group 45 Tobacco abuse |
| 32 | Dementia | P05, P20, P70, P71, P73 | Group 32 Dementia |
| 33 | Schizophrenia, retard mental | P72, P85, P80, P81, P85, P98 | Does not exist |
| 34 | Shortness breath, cough, COPD/ asthma  Pulmonary embolism | R02, 03, 04, 05, 07, 08, 09, 23, 25, 29, 71, 75, 76, 77, 79, 95, 96, 97, 98, 99  A92, K93 | Group 16 Asthma/COPD |
| 35 | Skin conditions (all without neoplasm) | All codes from S01 to S76 and from S85 to S99 | Group 37 Psoriasis |
| 36 | Eating problems and disorders, obesity, overweight | T02, T03, T04, T07, T08, T11, T82, T83, P86 | Group 10 Obesity |
| 37 | Thyroid, gout and metabolic disorders | T80, T81, T85, T86, T99 | Group 8 Thyroid dysfunction  Group 11 Purine/Pyrimidine metabolism disorders/Gout |
| 38 | Diabetes | T87, T89, T90 | Group 6 Diabetes mellitus |
| 39 | Urological disturbance and incontinence | U01, U02, U04, U05, U06, U07, U08, U13, U29, U71, U85, U99 | Group 33 Urinary incontinence |
| 40 | Kidney | U14, U88, U95 | Group 20 Renal insufficiency  Group 34 Urinary tract calculi |
| 41 | Female genital pain and menstrual problems | X01, X02, X03, X04, X05, X06, X07, X08, X09, X13, X14, X15, X16, X17, X84, X85, X86, X87 | Group 17 Non-inflammatory gynecological problems |
| 42 | Breasts | X18, X19, X20, X21, X22, X88, X99 | Does not exist |
| 43 | Menopausal | X11, X12 | Does not exist |
| 44 | Male genital pain | Y01, Y02, Y03, Y04, Y05, Y10, Y81, Y82, Y83, Y84 | Does not exist |
| 45 | Benign prostatic hypertrophy, prostate other | Y06, Y85 | Group 12 Prostatic hyperplasia |
| 46 | Sexuality | P07, P08, P09, Y07, Y08 | Group 43 Sexual dysfunction |
