# Supplementary material for: Multimorbidity: can general practitioners identify the health conditions most important to their patients? Results from a national cross-sectional study in Switzerland
Source: BMC Fam Pract. 2018 May 17;19:66. doi: 10.1186/s12875-018-0757-y (PMC5960174; doi:10.1186/s12875-018-0757-y)
Supplement: Supplementary file 2 — Table S2. a Agreement as per the 46 groups of conditions (N = 572). Table S2. b Agreement as per the chapters of the ICPC-2 classification (N = 585). (DOCX 20 kb) [file 12875_2018_757_MOESM2_ESM.docx]

Supplementary Table 2a -Agreement as per the 46 groups of conditions (N = 572)

| **Group of conditions** | **No. of times condition reported most important by patients** | **No. of disagreements** | **No. of agreements** | **Percentage of agreements** |
| --- | --- | --- | --- | --- |
| 1 | 19 | 16 | 3 | 15.8 |
| 2 | 2 | 2 | 0 | 0.0 |
| 3 | 29 | 11 | 18 | 62.1 |
| 4 | 1 | 0 | 1 | 100.0 |
| 5 | 4 | 2 | 2 | 50.0 |
| 6 | 11 | 4 | 7 | 63.6 |
| 7 | 3 | 1 | 2 | 66.7 |
| 8 | 10 | 6 | 4 | 40.0 |
| 9 | 2 | 1 | 1 | 50.0 |
| 10 | 3 | 2 | 1 | 33.3 |
| 11 | 3 | 2 | 1 | 33.3 |
| 12 | 8 | 4 | 4 | 50.0 |
| 13 | 10 | 7 | 3 | 30.0 |
| 14 | 15 | 4 | 11 | 73.3 |
| 15 | 9 | 8 | 1 | 11.1 |
| 16 | 11 | 9 | 2 | 18.2 |
| 17 | 10 | 4 | 6 | 60.0 |
| 18 | 121 | 39 | 82 | 67.8 |
| 19 | 70 | 40 | 30 | 42.9 |
| 20 | 8 | 6 | 2 | 25.0 |
| 21 | 15 | 3 | 12 | 80.0 |
| 22 | 5 | 3 | 2 | 40.0 |
| 23 | 3 | 1 | 2 | 66.7 |
| 24 | 7 | 1 | 6 | 85.7 |
| 25 | 5 | 3 | 2 | 40.0 |
| 26 | 14 | 8 | 6 | 42.9 |
| 27 | 7 | 0 | 7 | 100.0 |
| 28 | 19 | 6 | 13 | 68.4 |
| 29 | 5 | 4 | 1 | 20.0 |
| 30 | 4 | 2 | 2 | 50.0 |
| 31 | 3 | 3 | 0 | 0.0 |
| 32 | 7 | 3 | 4 | 57.1 |
| 34 | 33 | 15 | 18 | 54.5 |
| 35 | 12 | 7 | 5 | 41.7 |
| 36 | 2 | 2 | 0 | 0.0 |
| 37 | 1 | 1 | 0 | 0.0 |
| 38 | 69 | 22 | 47 | 68.1 |
| 39 | 5 | 0 | 5 | 100.0 |
| 40 | 2 | 1 | 1 | 50.0 |
| 45 | 5 | 5 | 0 | 0.0 |

Supplementary Table 2b - Agreement as per the chapters of the ICPC-2 classification (N = 585)

| **Group of conditions** | **No. times condition reported most important by patients** | **No. of disagreements** | **No. of agreements** | **Percentage of agreements** |
| --- | --- | --- | --- | --- |
| A | 51 | 34 | 17 | 33.3 |
| B | 4 | 2 | 2 | 50.0 |
| D | 24 | 7 | 17 | 70.8 |
| F | 10 | 6 | 4 | 40.0 |
| H | 2 | 1 | 1 | 50.0 |
| K | 64 | 18 | 46 | 71.9 |
| L | 199 | 65 | 134 | 67.3 |
| N | 43 | 14 | 29 | 67.4 |
| P | 39 | 10 | 29 | 74.4 |
| R | 38 | 14 | 24 | 63.2 |
| S | 13 | 8 | 5 | 38.5 |
| T | 76 | 24 | 52 | 68.4 |
| U | 8 | 2 | 6 | 75.0 |
| X | 3 | 2 | 1 | 33.3 |
| Y | 11 | 5 | 6 | 54.5 |
